# Genetic diversity of *Aedes aegypti* in the central-upper Paraná Cross-Border axis in Paraguay

**DOI:** 10.17843/rpmesp.2022.392.10709

**Published:** 2022-06-30

**Authors:** Sady C. Britez, Leidi Herrera, María C. Ferreira, Leticia M. Rolón, Vanessa Ruiz, Nilsa González-Brítez

**Affiliations:** 1 Instituto de Investigaciones en Ciencias de la Salud, Universidad Nacional de Asunción, San Lorenzo, Paraguay. Universidad Nacional de Asunción Instituto de Investigaciones en Ciencias de la Salud Universidad Nacional de Asunción San Lorenzo Paraguay; 2 Facultad de Ciencias Exactas y Naturales, Universidad Nacional de Asunción, San Lorenzo, Paraguay. Universidad Nacional de Asunción Facultad de Ciencias Exactas y Naturales Universidad Nacional de Asunción San Lorenzo Paraguay; 3 Instituto de Zoología y Ecología Tropical, Facultad de Ciencias, Universidad Central de Venezuela, Caracas, Venezuela. Universidad Central de Venezuela Instituto de Zoología y Ecología Tropical Facultad de Ciencias Universidad Central de Venezuela Caracas Venezuela

**Keywords:** Aedes, Genetic Polymorphism, Random Amplified Polymorphic DNA Technique, Mosquito Vectors, Surveillance, Paraguay

## Abstract

**Objective:**

To determine the genetic diversity of Aedes aegypti in the Central-Alto Paraná cross-border road corridor of Paraguay, an area that has reports of dengue cases.

**Materials and methods:**

Twenty adult females were selected from hatching Ae. aegypti eggs from households geolocated in the departments of Alto Paraná, Caaguazú, Cordillera and Central, between 2018 and 2019. DNA was extracted from the tissue of females for amplifying their polymorphic patterns by random amplification of polymorphic DNA by PCR (RAPD-PCR), using primers H3 and B03 in order to identify genetic parameters of population diversity. The relationships between mosquito populations according to locality were observed by unpaired arithmetic mean analysis. We used DIVA-GIS 7.3.0 and MAXENT to analyze the suitable areas of actual and potential geographic distribution of these Ae. aegypti populations.

**Results:**

Forty loci were identified by RAPD-PCR profiling, with moderate gene differentiation (Gst = 0.12). The cross-border corridor presented bioclimatic conditions for the presence of variant populations of Ae. aegypti, with precipitation in the warmest quarter and mean temperature in the driest quarter being determinant in the distribution.

**Conclusions:**

There is evidence of moderate genetic diversity in Ae. aegypti populations from areas that have reported dengue cases in the cross-border road corridor linking the Central and Alto Paraná departments of Paraguay. The study of genetic variability of Ae. aegypti is very useful for entomo-epidemiological surveillance and evaluation of possible resistance to chemical control.

## INTRODUCTION

*Aedes aegypti* (Linnaeus, 1762) (Diptera: Culicidae) is the vector transmitting several arboviruses and is responsible for the circulation of several serotypes of dengue virus in sympatry with chikungunya and zika viruses in the Americas ^1^. Currently, these viruses, particularly dengue, have had a great impact on public health and on the loss of working hours. This loss translates into 70% of hospitalization costs and costs for treatment and subsidies that can reach up to 80% of the total cost ^1^. In the last quarter of 2020, 60,925 cases of dengue were reported in Paraguay, mostly in the capital district (Asunción) and the Central department ^2^.

*Ae. aegypti* females, which exercise hematophagy for oviposition, show increasing anthropophilic habits, associating their high population densities to the presence of water reservoirs in artificial or natural containers such as leaf axils, bamboo internodes and other phytotelmata plants (they accumulate water in their structures), which may be present in the home or peridomicile ^3^.

The population variability of *Ae. aegypti* is susceptible to climatic changes, mobility of human groups and selection by insecticides; knowledge of these factors contributes to the understanding of its population dynamics ^4^ ^-^ ^6^.

An alternative for the study of genetic analysis is the use of the molecular technique called random amplification of polymorphic DNA by PCR or Random Amplified Polymorphic DNA (RAPD), useful for the search of random repetitive sequences in genomic regions of high genetic variability, within and between populations, with a minimum amount of DNA and without a previous target sequence ^7^. The study of natural mosquito populations allows the determination of genetic variations existing in different geographical areas and provides information about gene flow events in geographically separated populations, providing data for surveillance and control programs in areas of high human dispersion and high trade in order to implement specific surveillance measures.

This study aimed to study the genetic diversity of *Ae. aegypti*, in populations of four departments of the cross-border axis constituted between the departments of Central, Cordillera, Caaguazú and Alto Paraná, Paraguay. The vectors were captured between 2018 and 2019, in localities with reports of dengue cases.

**KEY MESSAGES**

Motivation for the study: Knowledge of the biology of the *Ae. aegypti* mosquito, vector of arbovirosis agents in endemic countries, is important if its genetic diversity is studied according to bioclimatically defined areas.

Main findings: The results showed moderate genetic differentiation and gene migration that have probably maintained mosquito populations with genetic similarity. The Central-Alto Paraná cross-border axis showed favorable bioclimatic conditions modeled by DIVA-GIS and MAXENT for *Ae. aegypti*.

Implications: Entomo-epidemiological surveillance should be used in cross-border corridors because they are transit and trade routes that favor vector spread in Paraguay and neighboring countries.

## MATERIALS AND METHODS

### Study area

A descriptive study was carried out by using convenience sampling. 

We included specimens of *Ae. aegypti* from the departments of Central (city of San Lorenzo), Cordillera (city of Piribebuy), Caaguazú (city of Coronel Oviedo) and Alto Paraná (city of Hernandarias), all of which are adjacent areas and under the influence of the cross-border axis (route 2 and route 7) between Gran Asunción (Central) and Ciudad del Este (Alto Paraná). This corridor brings the border areas of Argentina closer to Greater Asunción and reaches Brazil through the Alto Paraná Department, which is characterized by high human mobility, transportation, migration and trade, with some records of autochthonous dengue outbreaks ^8^ (Table 1).

**Table 1:** Geoclimatic, sociodemographic and epidemiological localization of the areas of the Central-Alto Paraná transboundary axis, Paraguay, for collection of Aedes aegypti from dengue endemic areas.

Localities	Geographical coordinates	Climate	Population (inhabitants)	Commercial activity	Dengue cases (2018-2019)
Hernandarias (Alto Paraná)	25°22′00″ S 54°45′00″ O	T= 23.5 °C RH=100% TV= ± 4.2 °C pp=3 mm	79,735	Industry and services	10
Coronel Oviedo (Caaguazú)	25°26′53″S 56°26′28″O	T=22.9 °C RH=95% TV= ± 4.8 °C pp=15 mm	121,626	Livestock and agriculture	34
Piribebuy (Cordillera)	25°28′45″S 57°03′00″O	T=22 °C; RH=73% TV= ± 5.6 °C pp=161 mm	28,179	Tourism and crafts	117
San Lorenzo (Central)	25°20′35″S 57°30′34″O	T=22.9 °C RH=89.5% TV= ± 6 °C pp=99mm	258,919	Commerce and industry	1223

T: average annual temperature. RH: relative humidity. TV: annual temperature variation. pp: mean precipitation/month.

Source: Climate was taken from: https://es.weatherspark.com and dengue cases from the National Malaria Eradication Service (SENEPA) of Paraguay.

### Biological material

*Ae. aegypti* eggs were collected between May 2018 and May 2019; by placing two to three ovitraps in peridomiciliary areas of dwellings with favorable environmental characteristics for vector presence, such as humid and shaded areas, surrounding vegetation, etc. A single collection was carried out in 80 houses selected from a randomly chosen spot in each locality, in order to obtain natural groupings of individuals within each population ^9^. This collection yielded approximately 1500 to 2000 eggs per locality. The ovitraps were removed seven days after placement and transported to the laboratory until mosquitoes developed into adult form, which were maintained for 24 h at 78% relative humidity, 28 °C temperature and fed with sugar solution. The taxonomic classification of the species was based on morphology and taxonomic criteria ^10^ ^,^ ^11^.

Specimens of the Rockefeller reference strain of *Ae. aegypti* (highly inbred and with more than 25 years of laboratory rearing) maintained under the same conditions were used as controls.

### DNA extraction

We used 20 female individuals from each population, placing each one in a microcentrifuge tube to which 240 µL of 5% Chelex® resin and 40 µL of 0.1M NaCl 0.1M saline solution were added for subsequent maceration. The contents were incubated at 99 ⁰C for 10 min and then centrifuged for 15 min; 200 µL of the supernatant were taken and preserved at -20 °C until use.

### Amplification by RAPD-PCR

The amplification reaction was formulated using a final reaction volume of 20 µL, which contained 0.2 mM dNTPs, 1.2 mM MgCl_2_, 1U 1X buffer; 4.0 *p*mol of the random insertion primers H3 (5'CATCCCCCCCTG'3) and B03 (5'CATCCCCCCCTG'3) Macrogene® (the PCR reaction was developed with eight commercial oligonucleotides: OP01, OP02, OP13, OP14, OP16, A2, H3, B03; primers were selected based on their best reproducibility and presence of polymorphic products), 1.0 U of thermostable platinum DNA *Taq Polymerase platinum* enzyme (Taq DNA Polymerase, Invitrogen®) and 17.5 ng/µL template DNA. The amplification procedure was carried out using the PTC-100 thermal cycler (MJ Research®, MA, USA) under the following conditions: initial denaturation at 94 °C for 2 min, followed by 45 cycles with: denaturation at 93 °C for 1 min, hybridization at 35 °C for 1 min, extension at 72 °C for 2 min and a final extension at 72 °C for 5 min ^6^.

The amplification products were separated by electrophoresis in a 2% agarose gel with 1X TAE buffer, run at 80 V for 1 h 45 min. The gel was stained with ethidium bromide for visualization of the bands and identification of their size, which were contrasted with molecular markers of 100 bp (DNA Ladder from Promega®) and 200 bp (Hyperladder I Bioline®). For photodocumentation we used a UV transluminator, model Vilber E-box.

### Data analysis

The RAPD-PCR markers were analyzed under the assumption that they segregated following Mendelian ratios and that the gene frequencies obtained were in Hardy-Weinberg ratios ^12^. Allele frequencies were estimated under the criterion of the presence of bands of an allele, assuming that each band is the product of a dominant allele at a homozygous or heterozygous locus, where the absence of a band represents the homozygous recessive genotype. The scoring of these resulted in the percentages of polymorphic loci ^13^.

Genotypic diversity indices (Shanon); total gene diversity (Ht); gene diversity of individuals in relation to their specific population (Hs); the gene differentiation coefficient (Gst estimator) as an indicator of genetic structuring between subpopulations and the effective migration rate or gene flow (Nm) ^14^ were determined using the population genetics program POPGENE (version 1.3.2). We constructed an intrapopulation genetic relationship dendrogram using the unweighted pair group mean analysis (UPGMA) algorithm ^15^.

The actual and potential geographic distribution of suitable areas of occurrence of RAPD-PCR variants of *Ae. aegypti* was analyzed using DIVA-GIS 7.3.0 and MAXENT software. We used data on the presence of geolocated *Ae. aegypti* using a GPS-Garmin® GPSMAP 64sx series GPS device and uploaded to Excel software. The data were read in DIVA-GIS 7.3.0, in order to generate a raster map of the real distribution of the populations under study, according to the 19 bioclimatic variables of Global Climate Data-WorldClim (http://www.wordclim.org), in raster format, with interpolation of data such as diurnal and annual temperature ranges, isothermality, average temperatures of the coldest/hottest, driest/wettest quarter of the year, annual precipitation, precipitation of the wettest/driest month, precipitation of the driest/coldest or wettest/driest quarter compatible with the geography of Paraguay (resolution of 1 km^2^ at the equator), predictors for 50 years (1950-2000). MAXENT software was used to associate the georeferenced points (ASCII format) and the described bioclimatic variables (ASCII format) in runs of 10 models and 1000 iterations ^16^ ^,^ ^17^. This information was contrasted with bioecological data associated with temperature and humidity for *Ae. aegypti*.

### Ethical considerations

The work was approved by the Ethics Committee of the Research Institute of the National University of Asuncion, whose approval code was P36/2015. 

The placement of traps was conducted with prior consent of the owners, the insect samples used were the minimum necessary to achieve optimal results and were handled under biosafety criteria. The rights of authors and sources consulted were respected.

## RESULTS

**Figure 1:**
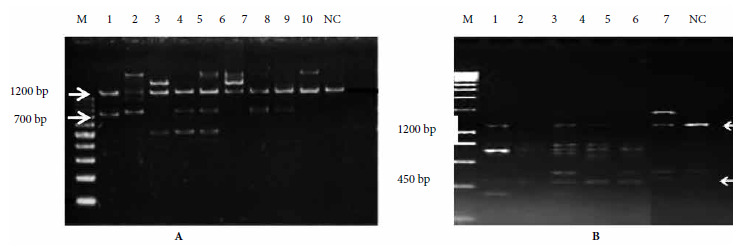
A) Band amplification patterns by random amplification of polymorphic DNA by PCR (RAPD-PCR) for the study of variability in natural populations of *Aedes aegypti* from the Central-Alto Paraná cross-border axis in Paraguay. B) Profiles of bands amplified with primer H3. White arrows indicate common band profiles corresponding to approximately 700 and 1200 bp (Lanes: 1-2 Hernandarias, 3-4 Caaguazú, 5-6 Piribebuy, 7-8 San Lorenzo, 9-10, Rockefeller [standard strain]), NC: negative control, M: molecular weight marker/100 bp DNA Ladder from Promega®. Profiles of bands amplified with primer B03. The white arrows indicate the common band profiles corresponding to approximately 450 and 1200 bp (lanes: 1-2 Piribebuy, 3-4 Hernandarias, 5-7 Caaguazú), NC: negative control, M: molecular weight marker/200bpb Hyperladder I Bioline®.

The collection provided 1500 to 2000 eggs per locality, from which we obtained the adults that provided the genetic material. The RAPD technique revealed the amplification of 40 bands (between 300 and 1700 bp), with primers H3 and B03, four of which corresponded to conserved fragments of 700 to 1200 bp for primer H3 and 450 to 1200 bp for primer B03; these were present in all individuals of the populations under study (Figure 1).

The analysis of the matrix of presence and absence of bands allowed us to estimate the mean values of Ht (total gene diversity) and Hs (gene diversity relative to the population), with which we attributed greater genetic diversity to that found between populations (interpopulation) and not within populations. The mean value obtained for the Gst=0.1231 index suggested moderate genetic differentiation ^18^ and the mean value of Nm inferred effective gene migration (Table 2), in line with the average of 15 polymorphic loci found in all the populations studied. The populations of Hernandarias and San Lorenzo showed the same percentage of intraspecific polymorphism (58.3%), while the populations of Caaguazú and the Rockefeller reference strain showed a percentage of intraspecific polymorphism of 62.5%. The highest percentage was found in the Piribebuy mosquito population (66.6%) (Table 3).

**Table 2:** Genetic diversity (Nei, 1973) intrapopulation of *Aedes aegypti* from four departments of the Central-Alto Paraná cross-border axis, analyzed by RAPD-PCR (random amplification of polymorphic DNA by PCR) independently of the study region.

Indices	H3 primer	B03 primer	Average values of the indices
I	0.3998	0.5173	0.4586
Ht	0.2457	0.3344	0.2900
Hs	0.2193	0.2879	0.2536
Gst	0.1073	0.1389	0.1231
Nm	4.1608 (%)	3.0990 (%)	3.6299 (%)

I: Shanon index. Ht: total gene diversity. Hs: gene diversity relative to the population. Gst: population gene structure estimator. Nm: gene flow.

The results come from the nested interpopulation matrix.

**Table 3:** Genetic diversity of Nei (1973) among *Aedes aegypti* populations from four departments of the Central-Alto Paraná cross-border axis analyzed by RAPD-PCR (random amplification of polymorphic DNA by PCR independently of the genetic marker).

Nei genetic diversity estimators (1973)				
Localities	I (Shanon)	h (Nei)	Polymorphic loci (%)	Number of polymorphic loci
Hernandarias	0.3135	0.2067	58.33	14
Caaguazú	0.3559	0.2400	62.50	15
Piribebuy	0.4055	0.2800	66.67	16
San Lorenzo	0.3494	0.2400	58.33	14

I: Shanon index. h: genetic diversity of Nei, 1973.

Results from an all-against-all interpopulation matrix without distinguishing between the molecular markers used.

**Figure 2:**
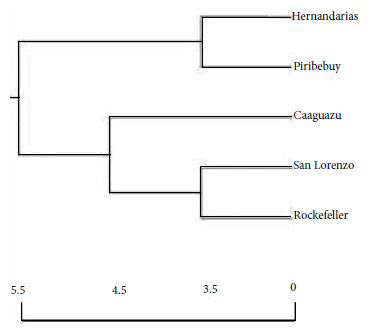
Dendrogram based on Nei’s genetic distance using the unpaired method of analysis of the arithmetic mean (UPGMA), Nei, 1978, constructed by analysis of band frequencies by random amplification of polymorphic DNA by PCR (RAPD-PCR), for *Ae. aegypti* populations from four localities in the Eastern Region of Paraguay and the reference Rockefeller strain (Pop Gene software).

The relationships between mosquito populations according to locality of origin, including the Rockefeller reference strain, revealed the existence of two groups (clusters), independently of the ecoregions; one group of the *Ae. aegypti* populations from Hernandarias, Piribebuy and another with the populations of San Lorenzo, Caaguazú and the Rockefeller reference strain. At the terminal point of the cluster, the Caaguazú populations separated from the rest of the populations from other regions of the axis and the San Lorenzo populations separated from those of the reference strain (Figure 2).

**Figure 3:**
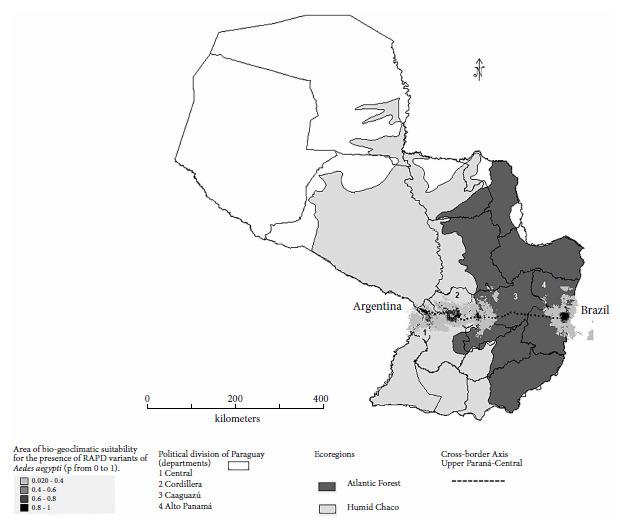
Areas of suitability for actual and potential spatial distribution of *Ae. aegypti* populations in the Central-Upper Paraná cross-border axis of Paraguay according to bioclimatic variables (DIVA-GIS and MAXENT; 10 percentile cut-off threshold). In light gray; the Humid Chaco ecoregion (Central and Cordillera) and in dark gray; the Atlantic Forest (Caaguazú and Alto Paraná).

The model of actual and potential spatial distribution of *Ae. aegypti* based on 19 bioclimatic variables (DIVA-GIS and MAXENT) confirmed the existence of suitable areas for vector establishment in 70% of the regions of the cross-border axis. The map shows the corridor between the ecoregions of the Humid Chaco (San Lorenzo-Central and Piribebuy-Cordillera) and the Atlantic Forest (Coronel Oviedo-Caaguazú and Hernandarias-Alto Paraná). The gray scale (0 to 1) indicates the probability of presence of the species (Figure 3).

The MAXENT model revealed that the bioclimatic variables suitable for the presence of *Ae. aegypti* were weighted 33.3% for precipitation in the warmest quarter (mean 184 mm/month), 13.2% for precipitation in the wettest month (mean 75 mm/month), and 13% for average temperature in the driest quarter (mean 26°C). The data were taken from the OGIMET Meteorological Information Service (https://www.ogimet.com).

The seasonality of temperature and isothermality followed each other in importance (approximately 7.4%), for the presence of the species. In other words, in the sampling areas, the seasonality of temperature was diverse according to the ecoregions, being 6 °C below the maximum temperature for the Humid Chaco and between 4 and 5 °C below the maximum temperature for the Atlantic Forest (OGIMET). On the other hand, the DIVA-GIS model revealed the presence of polar geographic points or “hot spots of presence” (black color) at the extremes of the cross-border axis, these being Asunción (near the border with Argentina) and Alto Paraná (near the border with Brazil) (Figure 3).

## DISCUSSION

Our results showed that the natural populations of *Ae. aegypti* collected in the Central-Alto Paraná cross-border axis in Paraguay had moderate genetic diversity indices, which seems to indicate that, along the cross-border axis, the population would be genetically stabilizing with a gene flow greater than 1 and medium weighting of the effect of migrations between localities.

Genetic diversity is conditioned, among other factors, by human inclusion or by the effects of natural environmental changes on the population dynamics of organisms ^19^, and allows the evaluation of the conditions of disease vectors, invasive species or the existence of improved and genetically modified varieties, as well as the intra- and inter-population gene flow of agents that have effects on public health ^20^.

These results are similar to the diversity indexes obtained by RAPD-PCR in six Brazilian states for *Ae. aegypti*, in which the Ht index was 0.390 with 27 polymorphic loci ^21^ and with 60 polymorphic loci, the Ht index was 0.388 ^22^; analogous results were reported in Mexico with 60 polymorphic loci, where the Ht index=0.34 ^12^ and in Puerto Rico this index was Ht= 0.35 with 57 polymorphic loci ^23^. These values revealed indices of moderate heterogeneity among the studied populations, in compliance with the classification criteria ^18^. The study populations showed moderate differentiation (Gst=0.107 for primer H3, and Gst=0.138 for primer B03) not dependent on geographic region. The short flight distance covered by the insect and the very low Gst indices suggest passive or forced dispersal between neighboring localities, favored by land transport and modulated by human commercial activity, as well as by accidental transport of eggs in artificial reservoirs; in this sense, some researchers have shown that there is a significant correlation between gene flow in *Ae. aegypti* and passive human transport ^24^. This type of migration would also explain the reduced variation between distant populations, which probably have not had enough evolutionary time to diverge, and their similarity results from the inertia of gene frequency over time ^21^ ^,^ ^25^ ^,^ ^26^.

Regarding the segregation cluster of the populations, constructed based on Nei’s genetic distance, we observed two similar groups, each with populations from both ecoregions and congruent with moderate genetic differentiation, with the Caaguazú population being the most divergent from the other populations. The similarity of the *Ae. aegypti* mosquito populations of San Lorenzo and the Rockefeller reference strain, which is highly inbred and laboratory-adapted, resembles artificial laboratory selection (Rockefeller strain) with the domiciliation of wild *Ae. aegypti* in highly anthropized regions (San Lorenzo), in this sense, under both conditions there is optimal growth, availability and frequency of blood intake, conferring “docility” to the behavior in space-time that intervenes in the selection processes ^27^.

The model of actual and potential spatial distribution of *Ae. aegypti* based on 19 bioclimatic variables (DIVA-GIS and MAXENT) confirmed the existence of suitable areas for the establishment of the vector in 70% of the regions of the cross-border axis (Argentina-Paraguay-Brazil), with the determining variables being, in order of importance, precipitation in the warmest quarter, precipitation in the wettest month, average temperature in the driest quarter, seasonality of temperature and isothermality.

The peri-urban areas near Coronel Oviedo (Caaguazú) do not appear to be suitable for establishment of the vector, probably because of the large distances between urbanized areas and access routes, the low population density and the high mobility of Amerindian family groups in the area, which would make it difficult for the vector to establish itself perennially, even when climatic conditions are favorable. In other countries, the distribution of *Ae. aegypti* has been reported in indigenous communities, where there are endemic areas of virus transmission and health emergencies related to dengue; however, in Paraguay, there are very few records of dengue in native populations ^28^.

A limitation of this study is that the RAPD-PCR marker requires rigorous standardization to reduce the variation caused by the components of the reaction, and its dominant nature implies indirect estimates of genetic diversity. However, its simple, rapid and less expensive methodology allows the detection of polymorphisms without sequencing or characterizing the genome of interest by analyzing several loci at the same time.

This study is the first to address a genetic study with natural populations of *Ae. aegypti* in Paraguay, which allowed us to approximate what would occur in a road axis of great commercial importance (land connection between Asunción, capital of Paraguay, and the border with Brazil, Alto Paraná), where *Ae. aegypti* is established; these dynamics should be monitored, as these are regions of high anthropic activity with tourist and commercial areas that establish entry and exit points between countries, thus favoring the dissemination of the transmitting agent, depending on the appearance of cases and the selection of subpopulations resistant to chemical control.

This study showed that there is moderate genetic diversity in *Ae. aegypti* populations from areas with records of dengue cases located in the cross-border road corridor linking the Central and Alto Paraná departments of Paraguay. Interpopulation diversity was greater than intrapopulation diversity, which leads us to assume that there are frequent but moderate migrations of the vector, conditioned by suitable bioclimatic factors that contribute to this differentiation. As a recommendation, we it is considered necessary to include other markers, such as mitochondrial genes that would allow us to continue with the assignment of haplotypes through reference tests such as the sequencing of the genome of *Ae. aegypti* in Paraguay, with the aim of better understanding its biodiversity and/or local migration routes to stratify risk areas in order to define better vector control strategies and methods to ensure adequate entomological surveillance.
