# Supplementary material for: Genetic diversity of Aedes aegypti in the central-upper Paraná Cross-Border axis in Paraguay
Source: Rev Peru Med Exp Salud Publica. 2022 Jun 30;39(2):170–7. doi: 10.17843/rpmesp.2022.392.10709 (PMC11397764; doi:10.17843/rpmesp.2022.392.10709)
Supplement: Translation

# Translation: Diversidad genética de *aedes aegypti* en el eje transfronterizo Central-Alto Paraná en Paraguay

**Authors:** Sady C. Britez, Leidi Herrera, María C. Ferreira, Leticia M. Rolón, Vanessa Ruiz, Nilsa González-Brítez

**Affiliations:** 1 Instituto de Investigaciones en Ciencias de la Salud, Universidad Nacional de Asunción, San Lorenzo, Paraguay.; 2 Facultad de Ciencias Exactas y Naturales, Universidad Nacional de Asunción, San Lorenzo, Paraguay.; 3 Instituto de Zoología y Ecología Tropical, Facultad de Ciencias, Universidad Central de Venezuela, Caracas, Venezuela.

**Keywords:** Aedes, Polimorfismo Genético, Técnica del ADN Polimorfo Amplificado Aleatorio, Mosquitos Vectores, Vigilancia, Paraguay

## Abstract

**Objetivos:**

Conocer la diversidad genética de *Aedes aegypti* en el corredor vial transfronterizo Central-Alto Paraná de Paraguay, con registros de casos de dengue.

**Materiales y métodos:**

Se seleccionaron veinte hembras adultas de la eclosión de huevos de *Ae. aegypti *procedentes de casas geolocalizadas en los departamentos de Alto Paraná, Caaguazú, Cordillera y Central, entre el 2018 y 2019. Se extrajo ADN del tejido de las hembras para amplificación aleatoria de sus patrones polimórficos mediante amplificación aleatoria del ADN polimórfico por PCR (RAPD-PCR), usando cebadores H3 y B03 a fin de conocer parámetros genéticos de diversidad poblacional. Las relaciones entre las poblaciones de mosquitos según la localidad fueron visualizadas mediante análisis no apareado de la media aritmética. Las áreas idóneas de distribución geográfica real y potencial de estas poblaciones de *Ae. aegypti *fueron analizadas mediante DIVA-GIS 7.3.0 y MAXENT.

**Resultados:**

Se identificaron 40 *loci* mediante perfiles RAPD-PCR, con diferenciación génica moderada (Gst = 0,12). El corredor transfronterizo presentó condiciones bioclimáticas para la presencia de poblaciones variantes de *Ae. aegypti,* siendo determinantes en la distribución la precipitación del trimestre más cálido y la temperatura media del trimestre más seco.

**Conclusiones:**

Se evidencia que existe diversidad genética moderada en las poblaciones de *Ae. aegypti* procedentes de zonas con registros de casos de dengue ubicadas en el corredor vial transfronterizo que une los departamentos Central y Alto Paraná de Paraguay. El estudio de variabilidad genética de *Ae. aegypti *es de gran utilidad para la vigilancia entomoepidemiológica y evaluación de posibles eventos de resistencia al control químico.

## INTRODUCCIÓN

*Aedes aegypti* (Linnaeus, 1762) (Diptera: Culicidae) es el vector transmisor de varias arbovirosis y es el responsable de la circulación de varios serotipos del virus dengue en simpatría con virus chikunguña y zika en las Américas ^1^. En la actualidad estas virosis, y en particular el dengue, han tenido gran impacto en la salud pública y en la pérdida de horas laborales. Esta pérdida se traduce en 70% de costos por hospitalización y costos por tratamiento y subsidios que pueden alcanzar hasta 80% del costo total ^1^. En el último trimestre de 2020, fueron registrados 60 925 casos de dengue en Paraguay, mayormente en el distrito capital (Asunción) y el departamento Central ^2^. 

Las hembras de *Ae. aegypti*, que ejercen la hematofagia para la oviposición, presentan hábitos antropofílicos crecientes, asociando sus altas densidades poblacionales a la presencia de depósitos de agua en recipientes artificiales o naturales como axilas de hojas, entrenudos de bambú y otras plantas fitotelmatas (acumulan agua en sus estructuras), que pueden estar presentes en el domicilio o el peridomicilio ^3^.

La variabilidad poblacional de *Ae. aegypti* es susceptible a cambios climáticos, movilidad de grupos humanos y selección por insecticidas, el conocimiento de estos factores contribuye a la comprensión de su dinámica poblacional ^4^ ^-^ ^6^.

Una alternativa para el estudio de análisis genético es el uso de la técnica molecular denominada amplificación aleatoria del ADN polimórfico por PCR o *Random Amplified Polymorphic DNA* (RAPD), de utilidad para la búsqueda de secuencias repetitivas aleatorias en regiones genómicas de alta variabilidad genética, dentro y entre poblaciones, con cantidad mínima de ADN y sin una secuencia diana previa ^7^. El estudio de poblaciones naturales de mosquitos permite determinar variaciones genéticas existentes en diferentes áreas geográficas y aporta información acerca de eventos de flujo génico en poblaciones separadas geográficamente, proporcionando datos para los programas de vigilancia y control en áreas de alta dispersión humana y elevado comercio a fin de implementar medidas específicas de vigilancia. 

En el presente trabajo se propuso estudiar la diversidad genética de *Ae. aegypti, *en poblaciones de cuatro departamentos del eje transfronterizo constituido entre los departamentos de Central, Cordillera, Caaguazú y Alto Paraná, de Paraguay. Los vectores fueron capturados entre los años 2018 y 2019, en localidades con registros de casos de dengue.

**MENSAJE CLAVE**

Motivación para realizar el estudio: conocer la biología del mosquito *Ae. aegypti, *vector de agentes de arbovirosis en países endémicos, tiene importancia si se estudia su diversidad genética con relación a áreas bioclimáticamente definidas.

Principales hallazgos: los resultados mostraron diferenciación genética moderada y migración de genes que probablemente han mantenido a las poblaciones de mosquitos con similaridad genética. El eje transfronterizo Central-Alto Paraná mostró condiciones bioclimáticas favorables modeladas por DIVA-GIS y MAXENT para *Ae. aegypti.*.

Implicancias: los corredores transfronterizos deben tener vigilancia entomoepidemiológica por ser vías de tránsito y de comercio, que favorecen la dispersión del vector en el Paraguay y países limítrofes.

## MATERIALES Y MÉTODOS

### Área de estudio

Se llevó a cabo un estudio descriptivo con un muestreo por conveniencia. Se incluyeron ejemplares de *Ae. aegypti* de los departamentos Central (ciudad de San Lorenzo), Cordillera (ciudad de Piribebuy), Caaguazú (ciudad de Coronel Oviedo), Alto Paraná (ciudad de Hernandarias), todas son zonas colindantes y bajo la influencia del eje transfronterizo (ruta 2 y ruta 7) entre Gran Asunción (Central) y Ciudad del Este (Alto Paraná). Este corredor aproxima las áreas limítrofes de Argentina con Gran Asunción y llega hasta Brasil a través del Dpto. de Alto Paraná, que se caracteriza por ser de alta movilidad humana, transporte, migración y comercio, con algunos registros de brotes de dengue autóctono ^8^ (Tabla 1).

**Tabla 1:** Localización geoclimática, sociodemográfica y epidemiológica de las áreas del eje transfronterizo Central-Alto Paraná, Paraguay, para colecta de *Aedes aegypti *de áreas endémicas de dengue

Localidades	Coordenadas geográficas	Clima	Población (habitantes)	Actividad comercial	Casos de dengue (2018-2019)
Hernandarias (Alto Paraná)	25°22′00″ S 54°45′00″ O	T= 23,5 °C HR=100% VT= ± 4,2 °C pp=3 mm	79 735	Industria y servicios	10
Coronel Oviedo (Caaguazú)	25°26′53″S 56°26′28″O	T=22,9 °C HR=95% VT= ± 4,8 °C pp=15 mm	121 626	Ganadería y agricultura	34
Piribebuy (Cordillera)	25°28′45″S 57°03′00″O	T=22 °C; HR=73% VT= ± 5,6 °C pp=161 mm	28 179	Turismo y artesanía	117
San Lorenzo (Central)	25°20′35″S 57°30′34″O	T=22,9 °C HR=89,5% VT= ± 6 °C pp=99mm	258 919	Comercio e industria	1223

T: temperatura anual promedio. HR: humedad Relativa. VT: variación anual de la temperatura. pp: precipitación media/mes.

Fuente: el clima fue tomado de: https://es.weatherspark.com y los casos de dengue del Servicio Nacional de Erradicación del Paludismo (SENEPA) de Paraguay.

### Material biológico

Los huevos de *Ae. aegypti* fueron colectados entre mayo del 2018 a mayo del 2019; a través de la colocación de dos a tres ovitrampas en áreas peridomiciliares de viviendas con características ambientales favorables para presencia del vector, tales como áreas húmedas y sombreadas, vegetación circundante, etc. Una única colecta se realizó en 80 viviendas seleccionadas a partir de un punto escogido al azar en cada localidad, con el fin de obtener agrupaciones naturales de individuos dentro de cada población ^9^. Dicha colecta permitió obtener aproximadamente 1500 a 2000 huevos por localidad. Las ovitrampas fueron retiradas a los siete días posteriores a la instalación, y transportadas al laboratorio, hasta la generación de mosquitos adultos, que fueron mantenidos por 24 h a 78% de humedad relativa, 28 °C de temperatura y alimentación con solución azucarada*.* La clasificación taxonómica de la especie se basó en su morfología y en claves taxonómicas ^10^ ^,^ ^11^.

Como control se incorporaron individuos de la cepa de referencia Rockefeller de *Ae. aegypti* (altamente endogámica y con más de 25 años de cría en laboratorio) mantenidos bajo las mismas condiciones.

### Extracción del ADN

Se usaron veinte individuos hembras de cada población, colocándose cada uno en un tubo de microcentrífuga al cual se le adicionó 240 µL de resina Chelex® al 5% y 40 µL de solución salina NaCl 0,1M para su posterior maceración. El contenido permaneció en incubadora a 99 ⁰C por 10 min para luego ser centrifugado por 15 min. Se tomaron 200 µL del sobrenadante y se preservaron a -20 °C hasta su uso. 

### Amplificación por RAPD-PCR

La reacción de amplificación se formuló utilizando un volumen final de reacción de 20 µl, el cual contenía 0,2 mM de dNTPs, 1,2 mM de MgCl_2_, 1U de *buffer* 1X; 4,0 *p*mol de los cebadores de inserción aleatoria H3 (5´CATCCCCCTG´3) y B03 (5´CATCCCCCTG´3) Macrogene® (la reacción de PCR fue desarrollada con ocho oligonucleótidos comerciales: OP01, OP02, OP13, OP14, OP16, A2, H3, B03; se seleccionaron los cebadores con base a su mejor reproducibilidad y presencia de productos polimórficos), 1,0 U de enzima ADN *Taq Polimerasa platinum* termoestable (*Taq *DNA Polymerase, Invitrogen®) y 17,5 ng/µL de ADN molde. El procedimiento de amplificación se realizó utilizando el termociclador PTC-100 (MJ Research®, MA, USA) bajo las siguientes condiciones: desnaturalización inicial a 94 °C por 2 min, seguido de 45 ciclos con: desnaturalización a 93 °C por 1 min, hibridación a 35 °C por 1 min, extensión a 72 °C por 2 min y una extensión final a 72 °C por 5 min ^6^. 

Los productos de amplificación fueron separados por electroforesis en gel de agarosa al 2% con *buffer *TAE 1X, corrida a 80 V por 1 h 45 min. El gel fue teñido con bromuro de etidio para visualización de las bandas e identificación de su tamaño, las cuales fueron contrastadas con marcadores moleculares de 100 pb. (DNA Ladder de Promega®) y 200 pb (Hyperladder I Bioline®). La fotodocumentación fue realizada a través de un transluminador UV, modelo Vilber E-box.

### Análisis de datos

Los marcadores RAPD-PCR se analizaron bajo la premisa de que se segregaron siguiendo las proporciones mendelianas y que las frecuencias génicas obtenidas estuvieron en proporciones de Hardy-Weinberg ^12^. Las frecuencias alélicas fueron estimadas bajo el criterio de presencia de bandas de un alelo, asumiendo que cada banda es el producto de un alelo dominante en un locus homocigoto o heterocigoto, donde la ausencia de banda representa el genotipo homocigoto recesivo. La puntuación de las mismas generó como resultado los porcentajes de *loci *polimórficos ^13^. 

Se determinaron los índices de diversidad genotípica (Shanon); la diversidad total de genes (Ht); la diversidad de genes de individuos en relación a su población especifica (Hs); el coeficiente de diferenciación génica (estimador Gst) como indicador de estructuración genética entre subpoblaciones y la tasa de migración efectiva o flujo de genes (Nm)^ (^ ^14^, usando el programa de genética poblacional POPGENE (versión 1.3.2). Se construyó un dendrograma de relación genética intrapoblacional, utilizando el algoritmo de análisis de la media de grupos de pares no ponderados (UPGMA) ^15^. 

La distribución geográfica real y potencial de las áreas idóneas de incidencia de variantes RAPD-PCR de *Ae. aegypti *fue analizada mediante *software* DIVA-GIS 7.3.0 y MAXENT. Se usaron los datos de presencia de *Ae. aegypti *geolocalizados mediante un dispositivo GPS-Garmin® serie GPSMAP 64sx y cargados al *software* Excel. Los datos fueron leídos en DIVA-GIS 7.3.0, para generación de mapa ráster de distribución real de las poblaciones en estudio, en función de las 19 variables bioclimáticas de *Global Climate Data-WorldClim* (http://www.wordclim.org), en formato ráster, con interpolación de datos tales como, rangos de temperatura diurna y anuales, isotermalidad, temperaturas promedio del trimestre más frio/caliente, más seco/húmedo del año, precipitación anual, precipitación del mes más húmedo/seco, precipitación del trimestre más seco/frío o más húmedo/seco compatibles con la geografía del Paraguay (resolución de 1 km^2 ^en el Ecuador), predictores para 50 años (1950-2000). El *software* MAXENT permitió asociar los puntos georreferenciados (formato ASCII) y las variables bioclimáticas descritas (formato ASCII) en corridas de 10 modelos y 1000 iteraciones ^16^ ^,^ ^17^. Esta información se contrastó con datos bioecológicos asociados a temperatura y humedad para *Ae. aegypti.*

### Consideraciones éticas

El trabajo fue aprobado por el Comité de Ética del Instituto de Investigaciones de la Universidad Nacional de Asunción, cuyo código de aprobación fue P36/2015. La colocación de trampas se realizó con previo consentimiento de los propietarios, las muestras de insectos utilizadas fueron las mínimas necesarias para lograr resultados óptimos y fueron manipuladas bajo criterios de bioseguridad. Se respetaron los derechos de autores y las fuentes consultadas.

## RESULTADOS

La colecta permitió obtener 1500 a 2000 huevos por localidad, de los cuales se obtuvieron los adultos que proporcionaron el material genético. La técnica de RAPD reveló la amplificación de 40 bandas (entre 300 y 1700 pb), con los cebadores H3 y B03, de las cuales cuatro correspondieron a fragmentos conservados de entre 700 a 1200 pb para el cebador H3 y de 450 a 1200 pb, para el cebador B03, presentes en todos los individuos de las poblaciones en estudio (Figura 1).

**Figura 1:** A) Patrones de amplificación de bandas por amplificación aleatoria del ADN polimórfico por PCR (RAPD-PCR) para el estudio de variabilidad en poblaciones naturales de* Aedes aegypti *del eje transfronterizo Central-Alto Paraná en Paraguay. B) Perfiles de bandas amplificadas con el cebador H3. Las flechas blancas indican los perfiles de bandas comunes correspondientes a 700 y 1200 pb aproximadamente. (Carriles: 1-2 Hernandarias, 3-4 Caaguazú, 5-6 Piribebuy, 7-8 San Lorenzo, 9-10, Rockefeller [cepa patrón]), CN: control negativo, M: marcador de peso molecular/100 pb DNA Ladder de Promega®. Perfiles de bandas amplificadas con el cebador B03. Las flechas blancas indican los perfiles de bandas comunes correspondientes a 450 y 1200 pb aproximadamente. (Carriles: 1-2 Piribebuy, 3-4 Hernandarias, 5-7 Caaguazú), CN: control negativo, M: marcador de peso molecular/200pb Hyperladder I Bioline®.

El análisis de la matriz de presencia y ausencia de bandas permitió estimar los valores medios de Ht (diversidad total de genes) y Hs (diversidad de genes relativa a la población), con los que se atribuyó mayor diversidad genética a la encontrada entre las poblaciones (interpoblacional) y no dentro de las poblaciones. El valor medio obtenido para el índice Gst=0,1231 sugirió diferenciación genética moderada ^18^ ^) ^y el valor medio de Nm infirió migración efectiva de genes (Tabla 2), en concordancia al promedio encontrado de 15 *loci* polimórficos en todas las poblaciones estudiadas. Las poblaciones de Hernandarias y San Lorenzo presentaron el mismo porcentaje de polimorfismo intraespecífico (58,3%), en tanto las poblaciones de Caaguazú y la cepa de referencia Rockefeller presentaron un porcentaje de polimorfismo intraespecífico de 62,5%. El mayor porcentaje lo presentó la población de mosquitos de Piribebuy (66,6%) (Tabla 3).

**Tabla 2:** Diversidad genética (Nei, 1973) intrapoblaciones de *Aedes aegypti* de cuatro departamentos del eje transfronterizo Central-Alto Paraná, analizadas por RAPD-PCR (amplificación aleatoria del ADN polimórfico por PCR*)* con independencia de la región de estudio

Índices	Cebador H3	Cebador B03	Valores medio de los índices
I	0,3998	0,5173	0,4586
Ht	0,2457	0,3344	0,2900
Hs	0,2193	0,2879	0,2536
Gst	0,1073	0,1389	0,1231
Nm	4,1608 (%)	3,0990 (%)	3,6299 (%)

I: índice de Shanon. Ht: diversidad total de genes. Hs: diversidad de genes relativa a la población. Gst: estimador de estructura génica poblacional. Nm: flujo de genes.

Los resultados provienen de la matriz interpoblacional anidada.

**Tabla 3:** Diversidad genética de Nei (1973) entre poblaciones de *Aedes aegypti *de cuatro departamentos del eje transfronterizo Central-Alto Paraná analizadas por RAPD-PCR (amplificación aleatoria del ADN polimórfico por PCR con independencia del marcador genético

Localidades	Estimadores de diversidad genética Nei (1973)			
	I (Shanon)	h (Nei)	Loci polimórficos (%)	Numero de loci polimórficos
Hernandarias	0,3135	0,2067	58,33	14
Caaguazú	0,3559	0,2400	62,50	15
Piribebuy	0,4055	0,2800	66,67	16
San Lorenzo	0,3494	0,2400	58,33	14

I: índice de Shanon. h: diversidad genética de Nei, 1973.

Los resultados provienen de una matriz interpoblacional todo contra todo sin discriminar marcador molecular utilizado.

Las relaciones entre las poblaciones de mosquitos según la localidad de procedencia incluyendo a la cepa patrón *Rockefeller*, revelaron la existencia de dos grupos (cl*ú*ster*)*, con independencia de las ecorregiones; un grupo entre las poblaciones de *Ae. aegypti *provenientes de Hernandarias, Piribebuy y otro con las poblaciones de San Lorenzo, Caaguazú y Cepa de referencia *Rockefeller*. En el punto terminal del cl*ú*ster, las poblaciones de Caaguazú se segregaron del resto de las poblaciones de otras regiones del eje y las poblaciones de San Lorenzo se segregaron con las de la cepa de referencia (Figura 2).

**Figura 2:** Dendrograma basado en distancia genética de Nei usando el método de análisis no apareado de la media aritmética (UPGMA), Nei, 1978, construido mediante análisis de frecuencias de bandas por amplificación aleatoria del ADN polimórfico por PCR (RAPD-PCR), para las poblaciones de *Ae. aegypti* procedentes de cuatro localidades de la Región Oriental del Paraguay y la cepa Rockefeller de referencia (*software* Pop Gene).

El modelo de distribución espacial real y potencial de *Ae. aegypti *en función de 19 variables bioclimáticas (DIVA-GIS y MAXENT) confirmó la existencia de áreas idóneas para el establecimiento del vector en un 70% de las regiones del eje transfronterizo. El mapa muestra el corredor entre las ecorregiones del Chaco Húmedo (San Lorenzo-Central y Piribebuy- Cordillera) y el Bosque Atlántico (Coronel Oviedo- Caaguazú y Hernandarias-Alto Paraná). La escala de grises (0 a 1), indica la probabilidad de presencia de la especie (Figura 3).

**Figura 3.** Áreas de idoneidad para la distribución espacial real y potencial de poblaciones de *Ae. aegypti* en el eje transfronterizo Central-Alto Paraná de Paraguay en función de variables bioclimáticas (DIVA-GIS y MAXENT; umbral de corte del percentil 10). En color gris claro; la ecorregión del Chaco Húmedo (Central y Cordillera) y en gris oscuro; el Bosque Atlántico (Caaguazú y Alto Paraná).

El modelo MAXENT reveló que las variables bioclimáticas idóneas para la presencia de *Ae. aegypti, *se ponderaron en forma contributiva en 33,3% para la precipitación del trimestre más cálido (media de 184 mm/mes), 13,2% para la precipitación del mes más húmedo (media de 75 mm/mes), y de 13% para la temperatura promedio del trimestre más seco (media de 26°C). Los datos fueron tomados del Servicio de Información Meteorológica OGIMET (https://www.ogimet.com). 

La estacionalidad de la temperatura y la isotermalidad se sucedieron en importancia (7,4% aproximadamente), para la presencia de la especie. Es decir que, en las zonas de muestreo, la estacionalidad de la temperatura resultó variante en función de las ecorregiones, siendo 6 °C por debajo de la máxima temperatura para el Chaco Húmedo y entre 4 y 5 °C por debajo de la máxima temperatura para el Bosque Atlántico (OGIMET). Por otro lado, el modelo DIVA-GIS reveló la presencia de puntos geográficos polares o «zonas calientes de presencia» (color negro) en los extremos del eje transfronterizo, siendo estos Asunción (próximo a la frontera con Argentina) y Alto Paraná (próximo a la frontera con Brasil) (Figura 3).

## DISCUSIÓN

Los resultados mostraron que las poblaciones naturales de *Ae. aegypti *colectadas en el eje transfronterizo Central-Alto Paraná en Paraguay, presentaron índices de diversidad genética moderada, lo que parece indicar que, a lo largo del eje transfronterizo, la población estaría estabilizándose genéticamente con un flujo de genes mayor a 1 y ponderación media del efecto de migraciones entre localidades.

La diversidad genética está condicionada entre otros factores por los efectos de cambios ambientales naturales o por la inclusión humana sobre la dinámica poblacional de organismos ^19^ y permite evaluar las condiciones de los vectores de enfermedades, especies invasoras o la existencia de variedades mejoradas y modificadas genéticamente, así como el flujo génico intra e interpoblacional de los agentes que tienen efectos en salud pública ^20^. 

Estos resultados son similares a los índices de diversidad obtenidos por RAPD-PCR en seis estados de Brasil para *Ae. aegypti ,* en el cual con 27 *loci* polimórficos el índice Ht fue 0,390 ^21^ y con 60 *loci* polimórficos, el índice Ht fue 0,388^ (^ ^22^ , resultados análogos fueron referidos en México con 60 *loci* polimórficos, donde el índice Ht=0,34 ^12^ y en Puerto Rico con 57 *loci* polimórficos este índice fue Ht= 0,35 ^23^. Estos valores revelaron índices de heterogeneidad moderada entre las poblaciones estudiadas, que acorde a los criterios de clasificación^ (^ ^18^. Las poblaciones de estudio mostraron diferenciación moderada (Gst=0,107 para el cebador H3, y Gst=0,138 para el cebador B03) no dependiente de la región geográfica. Las distancias cortas de vuelo del insecto e índices Gst muy reducidos sugieren una dispersión pasiva o forzada entre localidades colindantes, favorecida por el transporte terrestre y modulada por la actividad comercial humana, así como por el transporte accidental de huevos en depósitos artificiales; en tal sentido algunos investigadores demostraron que existe una correlación significativa entre el flujo de genes en *Ae. aegypti *y el transporte pasivo humano ^24^. Este tipo de migración adicionalmente explicaría la reducida variación entre poblaciones distantes, las cuales probablemente no han tenido suficiente tiempo evolutivo para divergir, y su similitud resulta de la inercia de la frecuencia de los genes a lo largo del tiempo ^21^ ^,^ ^25^ ^,^ ^26^ ^).^

En cuanto al *clú*ster de segregación de las poblaciones, construido en base a distancia genética de Nei, se observaron dos grupos de similaridad, cada uno con poblaciones de ambas ecorregiones; congruentes con la diferenciación genética moderada, siendo la población de Caaguazú, la más divergente de las demás poblaciones. La similitud de las poblaciones de mosquitos *Ae. aegypti *de San Lorenzo con la cepa de referencia Rockefeller, la cual es altamente endogámica y adaptada al laboratorio, asemeja la selección artificial de laboratorio (cepa Rockefeller) con la franca domiciliación de *Ae. aegypti *silvestre en las regiones altamente antropizadas (San Lorenzo), en tal sentido, en ambas condiciones se da un crecimiento óptimo, disponibilidad y frecuencia de ingesta sanguínea, confiriendo «docilidad» al comportamiento en espacio-tiempo que intervienen en los procesos de selección^ (^ ^27^. 

El modelo de distribución espacial real y potencial de *Ae. aegypti* en función de 19 variables bioclimáticas (DIVA-GIS y MAXENT) confirmó la existencia de áreas idóneas para el establecimiento del vector en un 70% de las regiones del eje transfronterizo (Argentina-Paraguay-Brasil), siendo las variables determinantes en orden de importancia, la precipitación del trimestre más cálido, la precipitación del mes más húmedo, temperatura promedio del trimestre más seco, estacionalidad de la temperatura y la isotermalidad. 

Las áreas periurbanas cercanas a Coronel Oviedo (Caaguazú), parecieran no mostrar idoneidad para el establecimiento del vector*,* probablemente influenciadas por las grandes distancias entre áreas urbanizadas y rutas de acceso, la baja densidad poblacional y la alta movilidad de grupos familiares amerindios presentes en la zona, lo cual dificultaría la instalación perenne del vector, aun cuando las condiciones climáticas favorezcan su presencia. En otros países se ha reportado la distribución de *Ae. aegypti *en comunidades indígenas, en las cuales hay zonas endémicas de transmisión del virus y emergencias sanitarias de infección por dengue, sin embargo en Paraguay, son muy escasos los registros de dengue en poblaciones originarias ^28^. 

Como limitación de este estudio se debe mencionar que el marcador RAPD-PCR requiere una rigurosa estandarización para reducir la variación provocada por los componentes de la reacción, y su condición de dominancia implica estimaciones de diversidad genética de manera indirecta. Sin embargo, su metodología sencilla, rápida y menos costosa permite detectar polimorfismos sin secuenciar o caracterizar el genoma de interés analizando varios *loci* a la vez. 

El presente trabajo es el primero que aborda un estudio genético con poblaciones naturales de *Ae. aegypti* en Paraguay, que permitió aproximar lo que ocurriría en un eje vial de gran importancia comercial (conexión terrestre de Asunción, capital del Paraguay y la frontera con Brasil, Alto Paraná), en donde surge el establecimiento de *Ae. aegypti, *cuya dinámica debe ser vigilada, por ser estas regiones de alta actividad antrópica con áreas turísticas y comerciales que establecen puntos de entrada y salida entre países, favoreciendo así la diseminación del agente transmisor, en función de la aparición de casos y en cuanto a la selección de subpoblaciones resistentes al control químico. 

Este estudio evidenció que existe diversidad genética moderada en las poblaciones de *Ae. aegypti* procedentes de zonas con registros de dengue ubicadas en el corredor vial transfronterizo que unen los departamentos Central y Alto Paraná de Paraguay. La diversidad interpoblacional fue mayor que la diversidad intrapoblacional, lo que nos lleva a suponer que existen migraciones frecuentes pero moderadas del vector, condicionados por factores bioclimáticos idóneos que contribuyen con dicha diferenciación. Como recomendación se considera necesario incluir otros marcadores, tales como los genes mitocondriales que permitirían continuar con la asignación de haplotipos a través de pruebas de referencia como la secuenciación del genoma del *Ae. aegypti* en Paraguay, con el objetivo de conocer mejor su biodiversidad y/o rutas de migración local que permitan estratificar áreas de riesgo para definir mejores estrategias de control vectorial y métodos que aseguren adecuada vigilancia entomológica.
